# A computational framework for the prioritization of disease-gene candidates

**DOI:** 10.1186/1471-2164-16-S9-S2

**Published:** 2015-08-17

**Authors:** Fiona Browne, Haiying Wang, Huiru Zheng

**Affiliations:** 1 Computer Science Research Institution, School of Computing and Mathematics, University of Ulster, Northern Ireland, UK

## Abstract

**Background:**

The identification of genes and uncovering the role they play in diseases is an important and complex challenge. Genome-wide linkage and association studies have made advancements in identifying genetic variants that underpin human disease. An important challenge now is to identify meaningful disease-associated genes from a long list of candidate genes implicated by these analyses. The application of gene prioritization can enhance our understanding of disease mechanisms and aid in the discovery of drug targets. The integration of protein-protein interaction networks along with disease datasets and contextual information is an important tool in unraveling the molecular basis of diseases.

**Results:**

In this paper we propose a computational pipeline for the prioritization of disease-gene candidates. Diverse heterogeneous data including: gene-expression, protein-protein interaction network, ontology-based similarity and topological measures and tissue-specific are integrated. The pipeline was applied to prioritize Alzheimer's Disease (AD) genes, whereby a list of 32 prioritized genes was generated. This approach correctly identified key AD susceptible genes: PSEN1 and TRAF1. Biological process enrichment analysis revealed the prioritized genes are modulated in AD pathogenesis including: regulation of neurogenesis and generation of neurons. Relatively high predictive performance (AUC: 0.70) was observed when classifying AD and normal gene expression profiles from individuals using leave-one-out cross validation.

**Conclusions:**

This work provides a foundation for future investigation of diverse heterogeneous data integration for disease-gene prioritization.

## Background

The rapid accumulation of high-throughput data along with advances in network biology have been fundamental in improving our knowledge of biological systems and complex disease. The emergence of network medicine [1] has explored disease complexity through the systematic identification of disease pathways and modules. Via the analysis of network topology and dynamics, key discoveries have been made including identification of novel disease genes and pathways, biomarkers and drug targets for disease [2]. Network theory is making important contributions to the topological study of biological networks, such as Protein-Protein Interaction Networks (PPIN) [3]. The study by Xu et al. [4] analyzed topological features of a PPIN and observed that hereditary disease-genes from the Online Mendelian Inheritance in Man (OMIM) database [5] have a larger degree and tendency to interact with other disease-genes in literature curated networks. Both Chuang et al. [6] and Taylor et al. [7] have indicated that the alterations in the physical interaction network may be a indicator of breast cancer prognosis. Goh et al. [8] demonstrated that the majority of disease genes are nonessential and located in the periphery of functional networks. Research by [9] discovered that genes connected to diseases with similar phenotypes are more likely to interact directly with each other.

Identification of candidate genes associated with physiological disorders are a fundamental task in the analysis of complex diseases [10]. Genome-wide association studies and linkage analysis have been pivotal in the identification of candidate genes, however, the large list of resultant genes returned are time-consuming and expensive to analyze [11]. The availability of high-throughput molecular interaction network provides and the application of network analysis tools such as clustering or graph partitioning have proved valuable in disease gene prioritization [12]. For instance, PPIN data integrated with genome-wide expression profiles using DNA arrays and/or next generation sequencing enabling the modeling of networks have aided our understanding of how biological networks operate. A number of computational approaches to prioritize candidate genes have been proposed including: ToppGene [13] and GeneWanderer [14] which rank candidate genes based on known associations with disease genes using diverse data sources and methodology. The study by Vanunu et al. [15] applied a diffusion- based method named PRINCE to prioritize genes in prostate cancer, AD and type 2 diabetes. Wu et al. proposed the resource AlignPI [16] which applied a network alignment approach predict disease genes. The algorithm VAVIEN [17] was also developed to prioritize disease genes based on topological features of PPINs.

These diverse studies confirm the importance and need of improving methods to integrate diverse 'omic' sources to uncover candidate disease genes in biological systems. To address this need, we have developed a prioritization pipeline, which integrates diverse heterogeneous information. We illustrate the implementation of this framework using Alzheimer's Disease (AD) as a Case Study. AD is the most common neurodegenerative disease which is both genetically complex and heterogeneous. Pathological characteristics of AD include presence of amyloid peptide plaques, mature senile plaques and neurofibrillary tangles and loss of neurons in conjunction with the presence of oxidative stress [18]. AD can be divided into two categories early onset AD (EOAD) (patients < 65) and late onset AD (LOAD) (patients > 65). A set of gene mutations including APP, PSEN1 and PSEN2 involved in the amyloid beta and tau pathways have been associated with hereditary AD. Using genome-wide association studies, Lambert et al. [19] identified the gene encoding APOE in LOAD as a risk factor along with 11 new loci. Furthermore, studies have suggested that AD is a multifactorial disease in which many pathways are involved. This highlights the progress, which has been made in determining the genetic underpinnings of AD. However, there is further need for an understanding of AD mechanisms to develop more specific diagnostic tests and novel drug therapies to target this disease.

Our proposed pipeline integrates AD gene-expression and network data along with ontology-based semantic similarity, topological information and tissue information. The integration of biological data such as semantic similarity which is independent of the gene expression profiles and PPIN used to obtain the significant hubs is advantageous in providing objective prioritization criteria. To evaluate the perfromance of our proposed method we (1) train an Random Forest classifier using features generated using the prioritized gene candidates to predict AD sample outcome using leave-one out cross-validation (LOOCV); (2) perform enrichment analysis and (3) compare the candidate gene list to a manually curated reference dataset of verified known and susceptible AD disease genes. Furthermore, we investigate the tissues in which AD candidate disease-genes are expressed through incorporation of tissue-specific expression data.

The remainder of the paper is organized as follows, in Section 2 the integrative framework is described along with details on datasets and PPINs used in the analysis. Section 3 provides a summary of the results obtained and conclusions along with future work is presented in Section 4.

## Methods

### Prioritization Workflow

**Figure 1:**
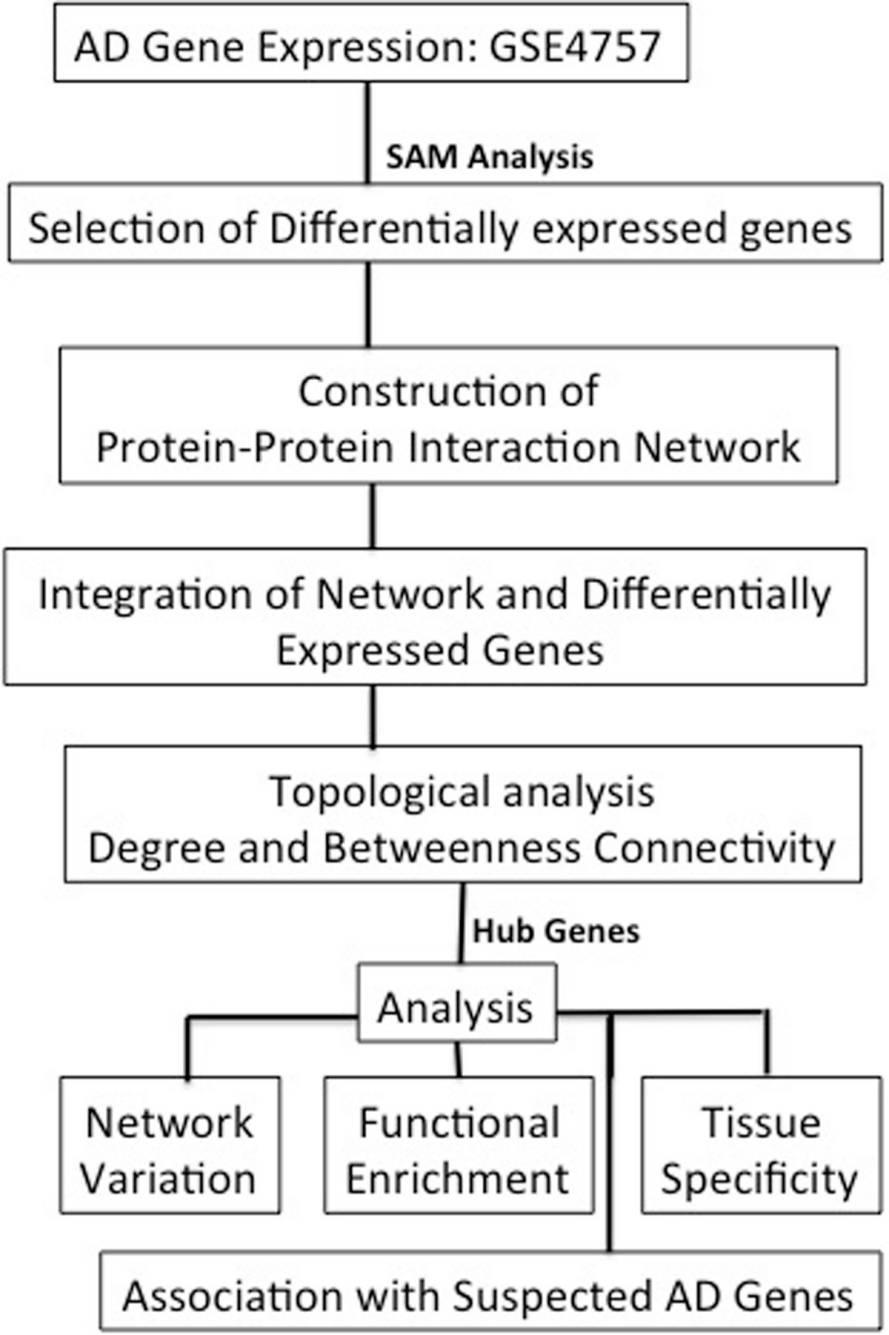
****Overview of Computational Framework to Prioritize Disease Gene Candidates****.

The schematic workflow of our computational integrative approach for disease gene prioritization is shown in Figure 1. Firstly, PPIN and gene-expression are integrated to provide an initial list of prioritized genes. Secondly, we incorporate additional data in the form of network topology and ontology-based semantic similarity to provide further prioritization. This list of genes is then evaluated using enrichment analysis and measurement of tissue specificity.

### AD gene expression data

Human AD gene expression data was obtained from the Gene Expression Omnibus (GEO) database. (http://www.ncbi.nlm.nih.gov/geo/). The selected profile GSE4757 was generated using the platform GPL570: Affymetrix Human Genome U133 Plus 2.0 Array. The study by Dunckley et al. [20] examined the transcriptome of entorhinal neurons from six cortical areas with or without neurofibrillary tangles (a histopathology feature of AD) using Laser capture microdissection. The dataset consists of gene expression profiles of NFT-bearing entorhinal cortex neurons from 10 mid-stage AD patients (Disease) compared with 10 histopathologically normal neurons (Control) from the same patients and brain region. These represent the different stages of AD according to the pattern of disease spread. Using the MAS5.0 function in R the CEL files were firstly normalized. Probes in expression profile were then mapped to corresponding NCBI Gene IDs. The average expression value was calculated in cases where the Gene ID related to more than one probe resulting in 20,539 unique Gene IDs.

A total of 10,106 significant genes were obtained using the significance analysis of microarrays (SAM) [21] technique, a regularized t-test approach, using the false discovery rate (FDR = 0.98). Differentially expressed (DE) genes are genes whose expression levels are significantly different between two groups of experiments.

### Human protein-protein interaction network

The PPIN was constucted using data from literature curated sources along with the recent Y2H screening by Rolland et al. [22]. The literature based dataset consists of 11,045 binary human protein pairs extracted from seven publically available databases including BioGRID [23], DIP [24], Biomolecular Interaction Network Database (BIND) [25], HPRD [26], InACT [27], Protein Data Bank (PDB) [28] and Molecular INTeraction database (MINT) [29]. The protein pairs were filtered on evidence where pairs that are only supported by two or more pieces of evidence are included. Protein pairs were also obtained from Y2H experimentation whereby 15,517 opening read frames (ORFs) were systematically screened using the platform hORFeome v5.1 (Space II) resulting in 13,944 pairwise interactions [22]. Both these data are integrated to produce a PPIN consisting of 7,783 nodes and 22,621 protein pairs.

### Toplogical analysis

AD gene expression data was mapped to the PPIN via NCBI geneIDs using Cytoscape version 3.2.1 [30]. This resulted in an AD disease specific network consisting of 5,457 nodes and 10,852 protein pairs. Hub genes were defined based on the measurement of network topological features (1) degree and (2) betweenness centrality using Cytoscape version 3.2.1 [30].

### Degree connectivity

Degree is a measure of the number of edges that connects a node. Genes with a high degree of connectivity within a network have large numbers of interacting partners. In PPINs it has been observed that genes with high degrees of connectivity are more likely to be essential as genes. Furthermore, many interacting partners in a network tend to be involved in important cellular processes [1]. Using this assumption, the top genes with the highest degree distribution were selected as hub proteins. This approach has previously been applied by Taylor et al. [7]. The degree cut-off threshold for selecting hubs is defined as:

(1) $$AVG+2*{\left(Std\right)}_{}$$

where *AVG *is the average degree across all DE genes in the PPINs and *Std*, the standard deviation [31].

#### Betweenness centrality

Betweenness is a topological feature of a network measuring information flow through the network. In biological networks, betweenness measures the paths through which signals can pass through the interaction network. Yu et. al. [32]. identified betweenness as an important topological property of a network where nodes with high betweenness control most of the information flow. The betweenness centrality *C_b_*(*n*) of a node *n *is computed as follows:

(2) $$C_{b}\left(n\right)=\underset{s\neq n\neq t}{\sum }\left(\theta_{st}\left(n\right)/\theta_{st}\right)$$

where *s *and *t *are nodes in the network different from *n, θ_st _*denoting the number of shortest paths from *s *to *t*, and *θ_st_*(*n*) is the number of shortest paths from *s *to *t *that *n *lies on. Betweenness centraility is calculated in Cytoscape. Using the node betweenness distribution, genes located in the top 50% are selected as hub genes.

### Network variation of hub genes

For each hub protein in the PPIN the average of Pearson correlation coefficients (PCC) between the hub and each of its respective partners was calculated for both disease and control groups. This method has previously been applied by Taylor et al. [7] to measure network variations among candidate genes and their interacting genes. To determine if interactions are varied, the difference of AD gene expression correlations of PPIs in disease and control samples is calculated. The average hub difference (*AvgPCC*) off correlation (Pearson's correlation co-efficient (PCC)) values between the disease and control groups was calculated as follows:

(3) $$Avg\text{P}CC=\frac{\sum_{i=1}^{n}\left(D_{i}-C_{i}\right)}{n}$$

where *D_i _*and *C_i _*represent the correlations of a hub and its interactors for the disease and control groups respectively and *n *the number of *i *interactors for a given hub.

Genes that are significantly different between the disease and control groups were selected as follows: (1) labels from the AD expression data were randomly assigned to either the disease or control group. (2) The *AvgPCC *was recalculated as *RandomPCC *by repeating the analysis defined in equation × 1000 times in order to calculate random distribution values. (3) *P *values for each hub was calculated by:

(4) $$\frac{\sum^{A}vgPCC\geq RandomPCC}{1000}$$

A network of significant hub genes was generated using significant cut-off threshold of *P *<= 0.05. *P *values are adjusted using Bonferroni correction. Random reassignment of the expression data was taken by randomly shuffling the expression data gene labels. This method of random reassignment retains the topological network structure of the interactome during the randomization.

### Ontology based semantic similarity

Genes involved in phenotypically similar diseases are often functionally related on the molecular level [33]. Based on this observation, the semantic similarity between hub genes and their interactors has been selected to analyze hub genes based on the Gene Ontology (GO) [34]. The GO is a controlled vocabulary describing the charateristics of gene products. Semantic similarity measures evaluate information two genes share. The functional similarity between two proteins is estimated using encoded information in the GO hierarchies. In this study Wang's measure of similarity [35] is applied to the Biological Process hierarchy. This measure determines the semantic similarity of two GO terms based on the locations of terms in the GO graph and their semantic relations with their ancestor terms. Given a GO term *A, T_A _*denotes the set of all its ancestor terms including term *A *itself. *S_A_*(*t*) can be defined as the contribution of a term $t\in T_{A}$ to the semantics of *A *based on the relative locations of *t *and *A *in the graph. Given GO terms A and B respectively, the semantic similarity between these two terms, *S_GO_*(*A,B*), is defined as:

(5) $${\text{S}}_{GO}\left(A,B\right)=\frac{\sum_{t\in T_{A}\cap T_{B}}\left(S_{A}\left(t\right)+S_{B}\left(t\right)\right)}{\sum_{t\in T_{A}}S_{A}\left(t\right)+\sum_{t\in T_{B}}S_{B}\left(t\right)}$$

As one gene may be annotated by many GO terms, similarity between two genes *Sim*(*G*_1_,*G*_2_), is then calculated by taking the average semantic similarity scores for all pairs of their associated terms. The similarity score can range between (0,1), whereby a value closer to 1 indicates close relatedness of the two genes in biological process. Wang's measure was implemented using the GOSemSim package in R [36], taking the median semantic similarity between a hub protein and its interactors.

### Construction of AD reference dataset

A reference dataset containing known and susceptible AD genes was constructed using the OMIM 'morbid map' table [5]. Known and recently discovered AD susceptibility genes in detailed in the study by Lamberet et al. [19] were also included. This resulted in a list of 52 AD related genes.

### Tissue specific gene expression data

Candidate genes were filtered using tissue-specific gene expression data retrieved from BioGPS [37] to determine if these genes are expressed in tissues where AD is observed including the tissue locations: whole brain and prefrontal cortex. This dataset contains the transcription levels of 84 human tissues and cell lines and was processed using the method described by Lopes et al. [38]. A list of 570 housekeeping genes were also included, obtained from [39].

### Classification of disease outcome

Construction of feature sets for individual samples were generated using the prioritized hub genes in order to classify sample outcomes applying the approach proposed by Taylor et al. [17]. Using the gene co-expression values for each sample in the AD dataset, the absolute difference in gene co-expression *CoeDiff *values of the prioritized genes and their interactors was calculated by:

(6) $$CoeDiff=\overset{n}{\underset{i=1}{\sum }}\left(I_{mi}-G_{i}\right)i=1$$

where the difference between the gene expression values of each prioritized gene *G *and its interactors *Im *is calculated. This was evaluated for all prioritized proteins across each sample in the AD dataset.

## Results and discussion

Using an integrative approach defined in the Method section, diverse heteregenous data obtained from AD gene expression, network topological analysis, along with semantic similarity and tissue-specific information are combined to to generate and analyze candidate AD genes.

### Selecting differentially expressed genes

The microarray GSE4757 was analyzed using Significance Analysis of Microarrays (SAM) to identify significantly expressed AD related genes in R using the SAM 5.0 package from [40]. A total of 10,107 significantly positive differentially expressed (DE) genes were observed from 20,539 genes in the AD microarray dataset based on the two class (disease and control) unpaired *t*-test, using the false discovery rate (FDR = 0.98). These DE genes were selected for the construction of the AD specific PPIN. An overview of the top 10 DE genes is presented in Table 1 along with the SAM score based on the *t*-statistic value.

**Table 1:** Overview of the top 10 differentially expressed genes obtained from the Alzheimer's microarray dataset using SAM analysis.

Gene Symbol	Gene Description	Score
Scd	stearoyl-CoA desaturase (delta-9-desaturase)	3.41
CCDC79	coiled-coil domain containing 79	3.10
STAU2	staufen, RNA binding protein, homolog 2 (Drosophila)	2.78
Hist1h2bc	histone cluster 1, H2bi; histone cluster 1, H2bg; histone cluster 1, H2be; histone cluster 1, H2bf; histone cluster 1, H2bc	2.72
HIST1H2BE	histone cluster 1, H2bi; histone cluster 1, H2bg; histone cluster 1, H2be; histone cluster 1, H2bf; histone cluster 1, H2bc	2.69
HIST1H2BF	histone cluster 1, H2bi; histone cluster 1, H2bg; histone cluster 1, H2be; histone cluster 1, H2bf; histone cluster 1, H2bc	2.56

HIST1H2BI	histone cluster 1, H2bi; histone cluster 1, H2bg; histone cluster 1, H2be; histone cluster 1, H2bf; histone cluster 1, H2bc	2.53
HIST1H2BG	histone cluster 1, H2bi; histone cluster 1, H2bg; histone cluster 1, H2be; histone cluster 1, H2bf; histone cluster 1, H2bc	2.52
DSTYK	dual serine/threonine and tyrosine protein kinase	2.50
Lrrc16a	leucine rich repeat containing 16A	2.47

### Construction of AD PPIN

The protein pairs in the human PPIN applied in this study have been derived from both small-scale studies described in literature and large-scale high-throughput Y2H experimentation. We integrate both these data in the construction of the PPIN to improve interactome coverage. Protein pairs obtained from small-scale studies are considered high quality, however, it has been observed by Rolland et al. [22] that their coverage of the interactome is limited to a narrow dense zone. Protein pairs obtained via high-throughput Y2H experimentation have demonstrated distributed homogeneously across the interactome [22].

AD gene expression data was firstly mapped to the PPIN via NCBI geneIDs using Cytoscape version 3.2.1 [30]. This network was further filtered to include only the 10,107 DE genes identified from the SAM analysis. The mapping of DE genes with the PPIN resulted in an AD disease specific network consisting of 3,795 nodes and 5,410 protein pairs.

### Identification of hubs through network topology analysis

Hub genes were firstly defined based on network topological features using Cytoscape version 3.2.1 [30]. The AD PPIN is represented as an undirected graphs, *G *= (*V,E*), whereby *V *represents a set of nodes (proteins) and E={(u,v)|u,v∈V}, the set of edges connecting the nodes. An overview of the global properties of the network is presented in Table 2. Using Cytoscape, a total of 174 hub genes were selecting based upon the topological analysis measures degree distribution and betweenness centrality. Genes with a high degree of connectivity and genes with low connectivity but high betweenness were selected using the cut-off thresholds defined in the Methods section. Table 3 presents the number of hubs obtained using these measures. These two measures have been selected as studies of model organisms have observed that proteins with high degree of connectivity tend to be encoded by essential genes [41]. Furthermore, detection of these genes leads to larger numbers of phenotypic outcomes compared to genes with lower connectivity [42]. However, not all disease genes in humans are essential genes. Goh et al. [8] found that non-essential disease genes tend to be tissue specific located at the functional periphery of the interactome and do not necessarily encode hubs. Both Yu et al. [32] and Joy et al. [43] demonstrated how nodes with a low degree of centrality but high betweenness are important in a network. Taking this into consideration, we also include betweenness as an indicator of centrality.

**Table 2:** Overview of the global properties of the AD specific PPIN.

Nodes	Edges	Clustering Co-Efficient	Avgerage Degree	Average Betweeness Centraility
3,795	5410	0.026	2.85	0.009

**Table 3:** Analysis of AD PPIN Topological Features to Identify Hub Genes.

	Number of Nodes
**High Betweenness** **(Bottlenecks)**	128
**Selected Hub Genes**	175

### Prioritization of hubs

#### Step 1: Integration of co-expression and PPIN

To further filter the list of 174 identified hub genes and select significant hubs we integrated the AD gene expression data with the PPIN. The network variation of the hub genes and their interactors was calculated using the *AvgPCC *equation defined in Methods. PCC values between the hubs and their interactors were calculated for both the disease and control groups. Significant hub genes were selecting using the Bonferroni corrected cut-off threshold of *P *< 0:05. A total of 22 genes were identified.

#### Step 2: Gene ontology semantic similarity analysis

The semantic similarity between hub genes and their interactors has also been selected to prioritize hub genes based on the Gene Ontology (GO). It has been observed that genes involved in phenotypically similar diseases are often functionally related on the molecular level [33]. In this study, the semantic similarity between a gene hub and it's interacting partners was performed using Wang's [35] measure of similarity detailed in equation 4. This was applied to the GO Biological Process hierarchy as a quantitative measure of functionality similarity between gene pairs using the R package GOSemSim [36]. To obtain the similarity value for the hub and all its interactors, the median similarity was taken across all protein pairs. The semantic similarity values obtained ranged between 0 and 1. The hub gene semantic similarity distribution was sorted in ascending order and hubs with GO semantic similarity greater than 0.5 selected. This resulted in a total of 10 prioritized hubs. Combining candidate genes output from analysis in Step 1 and Step 2 resulted in a list of 32 prioritized hub genes summarized in Table 4.

**Table 4:** List of significant hubs obtained from gene co-expression network analysis.

Approach	Number of Prioritized Hubs
Network Variation using PPIN and Gene Expression	22
Gene Ontology	10

### Evaluation

#### Functional annotation enrichment

GO and pathway enrichment analysis using the DAVID resource [44] was applied to investigate the biological implications of the prioritized hub gene list. Functional annotation was obtained by extracting the most over-representative GO terms (Biological Process, Cellular Component and Molecular Function) for the groups of genes under observation with respect to the whole genome taken as the reference background set (p-value <0.05). We applied this approach to measure if the prioritized hub genes are more enriched in GO terms or involved in pathways than what would be expected by chance. The number of GO terms and KEGG pathways are summarized in Table 5.

**Table 5:** Number of Enriched Gene Ontology Terms and KEGG Pathways.

	Number of Terms
GO Biological Process	135
GO Molecular Function	19
GO Cellular Component	17
KEGG Pathways	5

Enrichment analysis of the prioritized hub genes identified significant biological processes modulated in AD pathogenesis including: neuron differentiation, neuron projection morphogenesis, neuron projection development and regulation of neuron apoptosis. Furthermore, significant KEGG pathways including: the Wnt signaling pathway and the TGF-beta signaling pathway both of which have been implicated in neurodegenerative diseases [45,46] These results highlight the potential of this approach in using prioritized hubs for the prediction of AD biomarkers.

### Reference dataset comparison

The list of prioritized hub genes were compared to the reference dataset consisting of 52 AD related genes. A total of 9 AD susceptible genes from the list of hub genes identified were identified summarized in Table 6. Mutations in PSEN1 are the most common cause of early onset of AD. TRAF1, is a critical regulator of cerebral ischemia-reperfusion injury and neuronal death [47]. LZTS2 has shown associated with late onset AD [48].

**Table 6:** List of AD Susceptible Genes Idenfied from the Prioritized List of Gene Hubs.

Gene Symbol	Gene Name
LZTS2	leucine zipper, putative tumor suppressor 2
MTUS2	KIAA0774
TRAF1	TNF receptor-associated factor 1
FHL3	four and a half LIM domains 3
REL	v-rel reticuloendotheliosis viral oncogene homolog (avian)
CARD9	caspase recruitment domain family, member 9
TFCP2	transcription factor CP2
MID2	midline 2
KRT38	keratin 38

### Tissue analysis

The prioritized gene hubs were analyzed to determine if gene hubs are expressed in tissues in whereby by AD is observed including the prefrontal cortex and whole brain. Tissue specificity is an important component of network analysis as genetic diseases often target specific tissue(s). Therefore, perturbations of pathways or proteins may have differential effects among diverse tissues [49]. Using tissue specific expression data from BioGPS [37] along with housekeeping genes obtained from Lopes et al. [38], we identified that 4 of the prioritized gene hubs are located in the whole brain and/or the prefrontal cortex tissues.

#### Disease outcome classification

**Figure 2:**
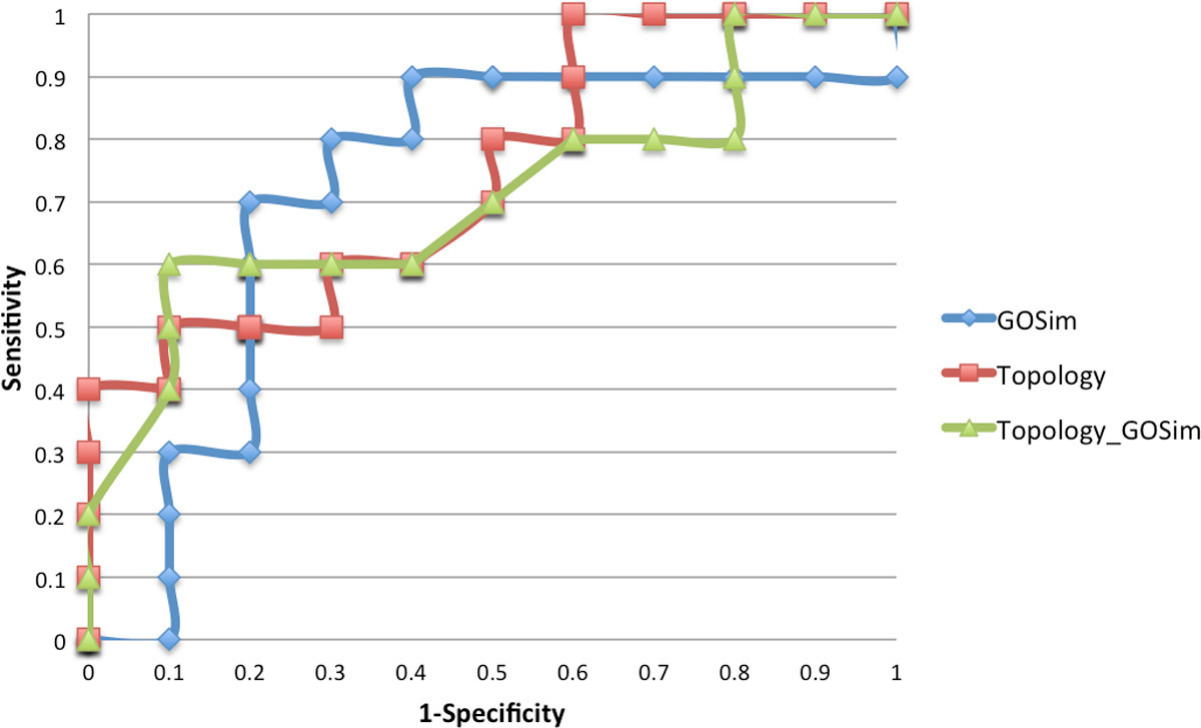
**ROC Curves illustrating the predictive performance of the Gene Hub Prioritization Approches**.

Using the prioritized gene-candidate list along with gene expression values from the GEO dataset GSE4757, we constructed a feature vector applying the approach described in Methods using Equation 6. These features measure the differences in absolute co-expression values between the prioritized genes and their interactors across each sample in the dataset. The aim of this approach is to determine if measured differences in co-expression values of prioritized genes and their interactors can predict sample outcome. A total of 32 feature values (obtained using each of the genes in the prioritized list) were generated for each instance in the AD dataset. The AD dataset has a total of 20 individual samples, 10 AD and 10 normal. We use these as labels when measuring the classification performance. Classification of sample outcome using this vector was performed using the Random Forest classifier [50] in the WEKA toolbox [51]. Leave one out cross validation (LOOCV) was implemented, whereby, for a dataset with *n *samples, *n *experiments are performed. Each experiment uses *n*-1 samples for training and the remaining sample for testing. The performance was assessed by a receiver operating characteristics (ROC) curve which plots the true positive rate against the false positive rate at various threshold settings. The area under the ROC curve (AUC) was estimated for each of the prioritization approaches described in Table 7. Reasonable AUC values were observed for the various prioritization approaches ranging from 0.73-0.70 as presented in Table 7 and illustrated using ROC curves in Figure 2. This analysis suggests that the integration of diverse data in the prioritization of protein hubs are indeed useful for sample outcome prediction. Interestingly, we can see a slight decrease in classification performance when gene hubs prioritized using topological features are integrated with gene hubs prioritized using GO semantic similarity. This is compared to using only the topological or GO semantic similarity prioritization approach only.

**Table 7:** Summary of Disease Outcome Classification using Prioritized Genes.

Priortization Approach	Description	Hubs	AUC Value
Topology	Hubs prioirtized using network topology methods defined in Step 1	22	0.74
GOSim	Hubs prioirtized using GO Semantic Similarity defined in Step 2	10	0.72
Toplogy_GoSim	Union of priortized hubs from Step 1 and Step 2	32	0.7

## Conclusions

In this study, we have proposed a novel computational framework that integrates diverse heterogeneous including gene expression, network topological features: degree and betweenness along with GO semantic similarity and tissue specificity information to prioritize and analyze disease-gene candidates. Our proposed pipeline provides the flexibility to integrate other heterogeneous data sources. To illustrate our approach, AD was applied as a Case Study. AD is a chronic neurodegenerative disorder characterized by a progressive decline in memory and cognitive abilities. It is estimated that 4-8% of the population over 65 years of age suffers from this disease with the rate of incidence increasing with age. Currently, only the symptoms of AD are treated with no effective therapeutic strategy available. Using our pipeline, 22 prioritized candidate AD disease genes were generated. Enrichment analysis revealed GO Biological Process terms were enriched by the prioritized gene-list such as regulation of neurogenesis and generation of neurons which are linked to AD pathogenesis. Analysis of KEGG pathways identified prioritized genes involved in pathways including Wnt signaling pathway and the TGF-beta signaling pathway. AD susceptible genes: PSEN1 and TRAF1 extracted from OMIN [5] and a recent study by Lambert et al. [19] were identified using the prioritization approach. A reasonable predictive performance (AUC: 0.70) was achieved when classifying AD and normal gene expression profiles from individuals using a feature set generated from the prioritized gene-list along with supervised classification using Random forest and LOOCV. These results demonstrate that the integration of PPINs along with disease datasets and contextual information is an important tool in unraveling the molecular basis of diseases. It is important to note that network based approaches for candidate disease gene priortization need to be viewed with care as the map of the binary human PPIN is still incomplete. However, coverage of the human interactome is increasing through systematic Y2H and transcriptional interaction screens. This increased coverage, quality, and diversity of human PPIN data will provide further opportunities for the molecular characterization and understanding of human disease [1].

## Competing interests

The authors declare that they have no competing interests.

## Authors' contributions

FB, HW and HZ conceived and designed the framework. FB performed the experiments and analyzed the data. FB, HW and HZ wrote the paper.
